# Plant-Derived Control of Postprandial Glycemia: α-Amylase, α-Glucosidase, and the Incretin-DPP-4 Interface

**DOI:** 10.3390/ph19101546

**Published:** 2026-09-30

**Authors:** Pirscoveanu Denisa Floriana Vasilica, Diana-Maria Trasca, Adina Maria Kamal, Renata Maria Varut, Romeo Popa, Pluta Ion Dorin, Dirnu Rodica, Maria Stoica, Coanca Staicu Cristina Teodora, George-Alin Stoica

**Affiliations:** 1 Department of Neurology, Faculty of Medicine, University of Medicine and Pharmacy of Craiova, 200349 Craiova, Romania; denisa.pirscoveanu@umfcv.ro; 2 Department of Internal Medicine, University of Medicine and Pharmacy of Craiova, 200349 Craiova, Romania; diana.trasca@umfcv.ro; 3 Research Methodology Department, Faculty of Pharmacy, University of Medicine and Pharmacy of Craiova, 200349 Craiova, Romania; coancastaicucristinateodora@gmail.com; 4 Drug Research Center, University of Medicine and Pharmacy of Craiova, 200349 Craiova, Romania; 5 Department of Pharmacology, University of Medicine and Pharmacy of Craiova, 200349 Craiova, Romania; romeo.popa@umfcv.ro; 6 Faculty of Medical and Behavioral Sciences, Constantin Brâncuși University of Târgu Jiu, 210185 Târgu Jiu, Romania; dorin.pluta@e-ucb.ro (P.I.D.); rodica.dirnu@e-ucb.ro (D.R.); 7 Department of Intensive Care and Anesthesia, Emergency County Hospital, 200642 Craiova, Romania; maria.stoica@umfcv.ro; 8 Department of Pediatric Surgery, Faculty of Medicine, University of Medicine and Pharmacy of Craiova, 200349 Craiova, Romania; alin.stoica@umfcv.ro

**Keywords:** postprandial glycemia, α-amylase, α-glucosidase, DPP-4, GLP-1, 1-deoxynojirimycin, Salacia, mulberry, natural products

## Abstract

Postprandial hyperglycemia can be modified before absorbed glucose reaches the circulation. Plant-derived compounds may slow starch hydrolysis through α-amylase and intestinal α-glucosidases, stimulate glucagon-like peptide-1 (GLP-1) release, or preserve incretin activity through dipeptidyl peptidase-4 (DPP-4) inhibition. This critical narrative review integrates these mechanisms around intestinal exposure, substrate selectivity, and human evidence. Flavonoids show structure-dependent and time-dependent inhibition of carbohydrate-hydrolyzing enzymes, but results vary markedly between yeast, rodent, and human enzyme systems. Iminosugar-rich mulberry preparations and thiosugar-containing Salacia extracts provide the strongest chemistry-to-clinic examples, including randomized trials that reduced postprandial glucose or HbA1c. Preclinical studies suggest that berberine may link bitter-taste-receptor-mediated GLP-1 secretion with local intestinal DPP-4 effects, whereas mangiferin, flavonol glycosides, anthocyanins, and food-derived peptides remain supported mainly by preclinical studies. In vitro potency alone is insufficient: meal composition, enzyme source, luminal concentration, metabolism, product standardization, gastrointestinal tolerance, and incretin measurements determine translational value. Candidate prioritization requires defined chemistry, achievable exposure, and product-specific human validation; acute effects do not establish durable benefit or human target engagement.

## 1. Introduction

Postprandial glucose excursions are determined by the amount and physical form of carbohydrate, gastric emptying, enzymatic hydrolysis, intestinal transport, incretin secretion, pancreatic insulin and glucagon responses, hepatic glucose handling, and peripheral glucose disposal. This sequence creates several opportunities for a locally acting intervention. A compound does not necessarily need high systemic bioavailability if it reaches intestinal enzymes or enteroendocrine cells at an effective concentration during a meal. Conversely, systemic claims such as DPP-4 inhibition require evidence that the active molecule or metabolite reaches the relevant compartment.

Three targets recur in the phytochemical literature. Salivary and pancreatic α-amylases initiate starch hydrolysis. Brush-border α-glucosidases, primarily maltase-glucoamylase and sucrase-isomaltase activities, release absorbable glucose from oligosaccharides and disaccharides. Dipeptidyl peptidase-4 (DPP-4) terminates the action of glucagon-like peptide-1 (GLP-1) and glucose-dependent insulinotropic polypeptide, while plant compounds can also stimulate GLP-1 secretion through nutrient-sensing or taste-receptor pathways. These mechanisms are related but not interchangeable. A candidate may inhibit carbohydrate hydrolysis without changing incretin exposure, increase GLP-1 secretion without inhibiting DPP-4, or affect both through altered nutrient delivery to the distal intestine.

The field is crowded with in vitro screening reports, yet only a small fraction reaches controlled human testing. The central translational problem is assay relevance. Yeast α-glucosidase differs from human intestinal enzymes; porcine pancreatic α-amylase is not identical to the human target; purified enzyme assays omit starch structure and food matrix; and colorimetric readouts can be distorted by strongly colored or reducing extracts; and docking cannot establish luminal potency. In the DPP-4 literature, inhibition of purified enzyme may not survive digestion, absorption, metabolism, or competition with related peptidases.

This review therefore asks a focused question: which plant-derived candidates connect defined chemistry, relevant target assays, plausible intestinal or systemic exposure, and human postprandial outcomes? Mulberry alkaloids and Salacia thiosugars are treated as anchor cases because they have progressed from mechanism to randomized trials. Flavonoids, berberine, mangiferin, anthocyanins, guava constituents, and food-derived peptides are evaluated against the same standard. The aim is not to rank herbs, but to define the evidence chain required for a credible postprandial glucose-control product.

Earlier reviews describe plant α-amylase inhibitors and polyphenol structure–activity relationships [1,2], while a recent review considers flavonoid effects on intestinal nutrient handling, gut hormones, and systemic metabolism [3]. The added contribution here is a common decision framework linking enzyme source, substrate, compartmental exposure, product identity, and the endpoint actually measured. It explicitly separates biochemical inhibition, GLP-1 secretion, DPP-4 target engagement, acute meal responses, and sustained HbA1c changes, and incorporates null clinical translation rather than ranking candidates by in vitro potency alone.

## 2. Literature Search and Evidence-Appraisal Framework

### 2.1. Review Design and Research Question

This study was designed as a structured critical narrative review addressing the translational evidence supporting natural-product modulation of postprandial glycemia through α-amylase, intestinal α-glucosidases, glucagon-like peptide-1 (GLP-1) secretion, and dipeptidyl peptidase-4 (DPP-4). The review specifically addressed which candidates demonstrate a coherent evidence chain linking chemical characterization, target-relevant activity, plausible intestinal or systemic exposure, and controlled human postprandial outcomes. The structured search supports a critical narrative synthesis; the review does not claim exhaustive systematic-review coverage or a pooled certainty-of-evidence assessment. The purpose was not to generate a pooled treatment-effect estimate, but to critically assess the progression of evidence from biochemical activity to clinically relevant target engagement.

### 2.2. Information Sources and Search Strategy

PubMed/MEDLINE, Scopus, and Web of Science Core Collection were searched. Three complementary search families were used to identify: (i) natural-product modulators of α-amylase and intestinal α-glucosidases; (ii) natural-product modulators of GLP-1 secretion and DPP-4 activity; and (iii) controlled human studies evaluating postprandial glycemic responses.

Search terms included combinations of “α-amylase”, “alpha-amylase”, “α-glucosidase”, “alpha-glucosidase”, “maltase-glucoamylase”, “sucrase-isomaltase”, “DPP-4”, “DPP-IV”, “GLP-1”, “glucagon-like peptide-1”, “incretin”, “postprandial glucose”, “postprandial glycemia”, “postprandial glycaemia”, “meal challenge”, “continuous glucose monitoring”, “flash glucose monitoring”, “plant”, “phytochemical”, “botanical”, “natural product”, “flavonoid”, “polyphenol”, “alkaloid”, “iminosugar”, “1-deoxynojirimycin”, “mulberry”, “Morus”, “Salacia”, “salacinol”, “kotalanol”, “berberine”, “berberrubine”, “palmatine”, “mangiferin”, “anthocyanin”, “guava”, “Psidium”, “food-derived peptide”, and “protein hydrolysate”. The search terms were organized into three concept blocks: target-related terms, natural-product terms, and human postprandial-outcome terms. The Boolean structure applied in each database was: (target-related terms AND natural-product terms) OR (human postprandial-outcome terms AND natural-product terms). In PubMed/MEDLINE, terms were searched within the title and abstract fields; in Scopus, within the title, abstract, and keyword fields; and in Web of Science Core Collection, within the Topic field. Database-specific syntax was applied, including [Title/Abstract] in PubMed/MEDLINE, TITLE-ABS-KEY in Scopus, and TS in Web of Science. No language filter was applied during the database searches, which covered the period from database inception to 19 August 2026. Eligibility was subsequently restricted to English-language publications during the screening process. The searches retrieved 82 records from PubMed/MEDLINE, 76 records from Scopus, and 62 records from Web of Science Core Collection, yielding 220 records before deduplication. After removal of 35 duplicate records, 185 unique records remained for title and abstract screening. The complete database-specific search strategies are provided in Supplementary Table S1.

### 2.3. Eligibility Criteria and Study Selection

English-language primary experimental and clinical studies were eligible when they evaluated a chemically identifiable natural product, phytochemical, botanical extract, standardized preparation, or natural-product-derived scaffold and provided target-relevant evidence for α-amylase, intestinal α-glucosidase, GLP-1 secretion, DPP-4 activity, or postprandial glycemic control. Eligible mechanistic studies included human or mammalian enzyme assays, kinetic studies, chemically characterized cellular models, intestinal models, pharmacokinetic or exposure studies, and in vivo experiments incorporating target-relevant measurements.

Human studies were eligible when they evaluated standardized meal or carbohydrate challenges, postprandial glucose or insulin responses, incremental area under the curve, continuous or flash glucose monitoring, incretin responses, DPP-4 activity, or longer-term glycemic outcomes using a defined intervention.

Studies based solely on molecular docking, total phenolic content, nonspecific antioxidant activity, or glucose lowering in animal models without target-relevant measurements were excluded from mechanistic inference. Editorials, case reports, conference abstracts or proceedings, non-English publications, and reports with insufficient intervention identity were excluded from the selected evidence corpus. Synthetic derivatives of natural-product scaffolds were retained only when they provided relevant structure–activity information and were not considered evidence of botanical-product clinical efficacy.

**Figure 1:**
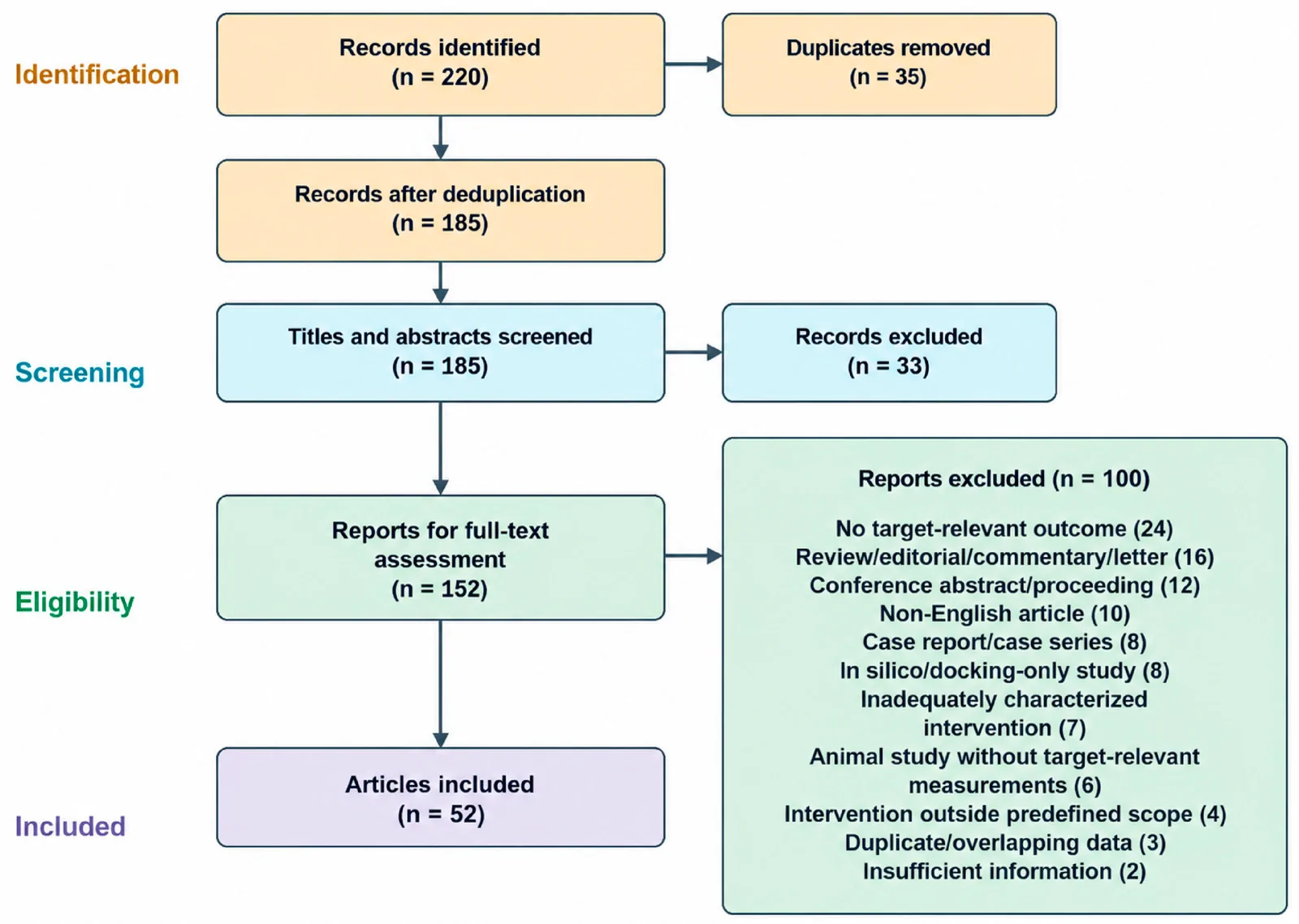
Literature-selection flow for the structured narrative review.

All retrieved records were exported to a reference-management database, and duplicates were removed before screening. Two review authors (P.D.F.V. and D.-M.T.) independently screened the titles and abstracts and subsequently assessed the potentially eligible full-text articles using separate screening records. Screening decisions were compared only after both reviewers had completed each stage. Disagreements were resolved through discussion and consensus. Any disagreement that could not be resolved through consensus was adjudicated by a third review author (R.M.V.). The selection comprised 220 identified records and 35 duplicate removals, leaving 185 records for title/abstract screening. After 33 exclusions, 152 reports entered full-text eligibility assessment; 100 were excluded, leaving 52 included articles (Figure 1). Exclusion reasons were no target-relevant outcome (*n* = 24), review/editorial/commentary/letter (*n* = 16), conference abstract/proceeding (*n* = 12), non-English article (*n* = 10), case report/case series (*n* = 8), in silico/docking-only study (*n* = 8), inadequately characterized intervention (*n* = 7), animal study without target-relevant measurements (*n* = 6), intervention outside the predefined scope (*(n* = 4), duplicate/overlapping data (*(n* = 3), and insufficient information *(n* = 2) (Figure 1).

### 2.4. Data Extraction and Evidence Classification

Data were extracted using a predefined standardized form including the following variables: study design, biological model or clinical population, natural-product identity, botanical source, plant part, extraction method or formulation, chemical marker or standardization, target, enzyme source, substrate, potency or kinetic parameters, administered dose or tested concentration, exposure information, comparator, carbohydrate or meal challenge, treatment duration, glycemic outcomes, insulin outcomes, GLP-1, GIP, and DPP-4 measurements, adverse events, funding information, and major methodological limitations.

Evidence was interpreted according to translational proximity using the operational categories in Table 1. These categories describe the type of support for a specific claim, not a validated risk-of-bias or certainty score. The highest weight was assigned to controlled human studies using chemically defined or standardized products with target-compatible outcomes, followed by human efficacy studies with incomplete target-engagement information, in vivo mechanistic studies, and target-relevant biochemical or cellular investigations. Exploratory evidence based primarily on yeast-enzyme screening, crude-extract screening, molecular docking, or indirect mechanistic inference was considered hypothesis-generating and was not regarded as sufficient to establish clinically relevant target engagement.

### 2.5. Critical Appraisal

Clinical studies were critically appraised according to study design, randomization and blinding where applicable, comparator selection, sample size, intervention standardization, outcome measurement, treatment duration, completeness of reporting, and potential sources of bias. For biochemical and cellular studies, methodological credibility was evaluated according to enzyme or model relevance, substrate appropriateness, use of positive and interference controls, chemical characterization, concentration–response assessment, kinetic characterization, biological replication, and exposure plausibility. For animal studies, particular attention was given to experimental-group comparability, target-relevant measurements, dose selection, and consistency between mechanistic and metabolic outcomes. Given the heterogeneity of the evidence base, no numerical quality score was assigned.

### 2.6. Evidence Synthesis

Because the included evidence spans biochemical, cellular, animal, pharmacokinetic, acute meal-challenge, and longer-term clinical studies, substantial heterogeneity was anticipated in biological models, enzyme sources, substrates, intervention composition, populations, doses, and outcome definitions. Therefore, quantitative meta-analysis was not planned. Evidence was synthesized qualitatively according to target mechanism, chemical identity, intended compartment of action, exposure plausibility, methodological credibility, and level of human translational support. Particular emphasis was placed on discrepancies between in vitro potency and in vivo or clinical outcomes, product-to-product non-equivalence, target-specific assay validity, and reproducibility across chemically comparable interventions. Prioritization considered chemical standardization, controlled-trial design and sample size, target-compatible outcomes, direct target-engagement measurements, independent replication, and duration. These are transparent interpretive criteria for this synthesis, not retrospectively claimed prespecified thresholds. Mulberry twig alkaloid tablets have the larger longer-duration trial program; Salacia has several acute trials across heterogeneous extracts. Neither ranking establishes a class effect or completes every step of the evidence chain (Table 2).

## 3. The Intestinal Pharmacology of Postprandial Glucose

Starch must be physically accessible before enzymatic hydrolysis can proceed. Granule structure, gelatinization, particle size, amylose-to-amylopectin ratio, protein and lipid interactions, and food viscosity all influence the rate at which amylases release shorter glucans. α-Amylase inhibition is therefore only one component of starch control. Polyphenols may also bind starch or alter matrix structure, meaning that an apparent reduction in hydrolysis can reflect enzyme inhibition, substrate sequestration, or both. Three flavanols, for example, delayed starch digestion through combined α-amylase inhibition and starch binding [10].

### 3.1. α-Amylase: Potency Is Substrate- and Time-Dependent

Plant-derived α-amylase inhibitors and the structure–activity relationships of dietary polyphenols have been extensively investigated in previous studies and reviews [1,2]. Flavonoids can inhibit human α-amylase through hydrogen bonding and aromatic interactions near the catalytic region. Work with human salivary α-amylase identified structural requirements including a conjugated π-system and hydroxyl groups positioned to interact with catalytic residues [4]. Broader kinetic studies showed that luteolin, hesperetin, and quercetin can produce mixed-type inhibition, whereas the apparent rank order depends on assay design and substrate [21]. A library of pancreatic α-amylase inhibitors likewise linked activity to the flavonoid substitution pattern rather than to total hydroxylation alone [22]. Early comparative work showing inhibition of both α-glucosidase and α-amylase by flavonoids remains a useful foundation, although enzyme source and substrate now require tighter control [23].

Binding kinetics can materially change an inhibition estimate. Gallated catechins interacted rapidly with human salivary and porcine pancreatic α-amylases, whereas non-gallated analogues required much longer incubation to approach maximal inhibition [11]. If a screen uses a preincubation period that does not occur during digestion, it may overestimate or underestimate meal-time efficacy. The substrate also matters: a small chromogenic analogue provides clean kinetics but does not reproduce enzyme access to cooked starch, starch-polyphenol binding, or diffusion within a food matrix.

Purified-enzyme inhibition establishes activity under defined biochemical conditions. Digestion of cooked starch or a food matrix adds substrate binding, accessibility, and contact-time constraints but remains preclinical. A controlled meal challenge tests the integrated human response and cannot, by itself, identify α-amylase as the causal target. These evidence types should therefore be reported separately [4,10,11].

Maximal α-amylase inhibition may be undesirable. If extensive starch reaches the colon, bacterial fermentation can produce bloating, flatulence, or diarrhea. A pharmacologically useful profile may therefore combine partial α-amylase inhibition with stronger, substrate-matched brush-border α-glucosidase inhibition, slowing glucose appearance without preventing eventual digestion. This principle is more informative than selecting the extract with the lowest nominal α-amylase IC50.

### 3.2. Brush-Border α-Glucosidases: The Enzyme-Source Problem

The term α-glucosidase often conceals major biological differences. Yeast enzymes are convenient for screening, but their active sites and inhibitor sensitivities differ from mammalian intestinal maltase-glucoamylase and sucrase-isomaltase. Compounds isolated from Vauquelinia corymbosa illustrate the problem: quercetin-3-O-(6″-benzoyl)-β-galactoside had IC50 values of 30 μM for yeast and 437 μM for rat intestinal α-glucosidase; the corresponding Ki values were 50 and 212 μM [12]. A low IC50 against yeast enzyme should therefore trigger confirmation, not a clinical claim.

Earlier enzyme-guided investigations examined *Tournefortia hartwegiana*, *Andrographis paniculata* and andrographolide, *Rheum emodi* constituents, and plants used in Lebanese traditional medicine [24,25,26,27]. Collectively, these reports demonstrate the usefulness of α-glucosidase and α-amylase assays for bioactivity-guided fractionation. They also expose a recurring limitation: activity against yeast enzyme, crude extracts, or a single chromogenic substrate cannot predict inhibition of human brush-border enzymes or the response to a mixed meal. Their strongest current role is therefore source discovery and chemical prioritization.

Target selectivity can be designed. In a comparative panel, the C2-C3 double bond and selected hydroxyl groups favored α-amylase inhibition, whereas a different arrangement supported α-glucosidase inhibition [13]. Salacinol analogues with extended side chains achieved higher potency against human intestinal maltase than the natural parent and in some cases matched or exceeded reference inhibitors [14]. These studies show how plant-derived scaffolds can inform medicinal chemistry even when the crude botanical product is not itself a drug candidate.

The approximately 15-fold IC50 difference in this within-study comparison illustrates source dependence, not a conversion factor from yeast to humans [12]. Potencies from different studies should remain stratified by enzyme source, substrate, and assay conditions; crude-extract concentrations in μg/mL are not directly comparable with purified-compound values in μM. Both maltase-glucoamylase and sucrase-isomaltase contribute overlapping intestinal activities, so a maltose challenge is not specific to only one enzyme complex.

### 3.3. Incretin Release and DPP-4: Direct and Indirect Intestinal Effects

GLP-1 is released from enteroendocrine cells after nutrient sensing and enhances glucose-dependent insulin secretion while suppressing glucagon and slowing gastric emptying. DPP-4 rapidly cleaves active GLP-1. Medicinal-plant-derived GLP-1 secretagogues have been previously reported and investigated as potential modulators of incretin signaling [28]. A plant product can increase active incretin exposure by stimulating secretion, reducing degradation, or changing the rate and site of nutrient delivery. α-Glucosidase inhibition itself may shift carbohydrate to more distal intestinal segments and alter incretin release. Direct L-cell stimulation should be demonstrated with receptor or signaling controls rather than inferred from a higher post-meal GLP-1 concentration.

DPP-4 is both a membrane-associated and soluble enzyme with substrates beyond incretins. Potency should be measured against human enzyme and interpreted with selectivity, cell-surface access, and exposure. Food-derived inhibitors may act locally before absorption, but systemic claims require stability through digestion and enough free compound in plasma or tissues. A comparison of digested proteins found no simple correlation between in vitro and in vivo DPP-4 inhibition, underscoring the influence of peptide release, intestinal passage, metabolism, and assay context [17].

Two representative studies illustrate why incretin measurements require mechanism-specific interpretation. Wild bitter gourd produced GLP-1-dependent hypoglycemic effects in a preclinical model [29], whereas a human cinnamon study jointly assessed gastric emptying, glucose, insulin, GIP, GLP-1, ghrelin, and satiety [15]. Such multi-endpoint designs are valuable because a botanical can change postprandial glucose through gastric emptying or carbohydrate delivery without directly inhibiting DPP-4. Active versus total GLP-1, sampling time, and an independent gastric-emptying measure are therefore essential.

## 4. Chemical Classes and Mechanistic Examples

Natural postprandial modulators span polyphenols, iminosugars, sulfonium thiosugars, isoquinoline alkaloids, xanthones, anthocyanins, and food-derived peptides. Their physicochemical properties imply different development strategies. Highly polar iminosugars and thiosugars can be effective in the intestinal lumen with limited systemic exposure. Polyphenols may bind enzymes and food macromolecules but are extensively metabolized. Alkaloids such as berberine can achieve high intestinal exposure despite low plasma concentrations. Peptides must survive digestion long enough to act locally or be released by digestion in an active form.

### 4.1. Flavonoids and Polyphenols

Flavonoid inhibition is structure-dependent rather than a generic antioxidant effect. Planarity, the C2-C3 double bond, the 4-carbonyl group, and hydroxyl positions determine enzyme binding, while glycosylation can either reduce access or create new interactions. Comparative experiments have produced partially different structure–activity rules because enzyme sources, substrates, incubation times, and analysis methods differ [4,13,21]. These disagreements are informative: they show that a universal flavonoid ranking is unlikely without a standardized assay panel.

DPP-4 inhibition by flavonoids is also chemically selective. In a purified-enzyme panel of 70 compounds, myricetin, hyperoside, narcissoside, cyanidin 3-O-glucoside, and isoliquiritigenin showed concentration-dependent inhibition with non-competitive or mixed kinetic behavior [18]. A separate study identified isorhamnetin-3-O-glucoside, cyanidin-3-O-glucoside, and isorhamnetin-3-O-rutinoside as potent inhibitors in purified and Caco-2-associated systems [19]. These are preclinical mechanistic findings, but glycoside stability, luminal concentrations, brush-border access, and selectivity against related peptidases must be established before therapeutic equivalence to pharmaceutical DPP-4 inhibitors is suggested.

Assay dependence is quantitatively substantial: Pan et al. reported IC50 values of 156.29 ± 1.18 μM for myricetin and 81.05 ± 4.14 μM for cyanidin 3-O-glucoside [18]. These values are assay-specific and do not establish attainable free concentrations or DPP-4 inhibition in humans.

### 4.2. Mulberry Iminosugars and 1-Deoxynojirimycin

Mulberry leaves and twigs contain iminosugar alkaloids, particularly 1-deoxynojirimycin (DNJ), that inhibit intestinal α-glucosidase. DNJ content offers a chemically meaningful standardization marker because it is linked to the proposed mechanism. In subjects with impaired glucose metabolism, a DNJ-enriched mulberry leaf extract reduced postprandial glucose and insulin responses after a rice challenge in a randomized crossover study [7]. Dose-finding work in obese people with borderline diabetes further evaluated glycemic profiles and tolerability of mulberry leaves standardized by DNJ [30].

Mulberry research also demonstrates why product identity must remain precise. Leaf extracts, twig alkaloid tablets, fruit extracts, teas, and combination products are not interchangeable. Their DNJ content, accompanying polyphenols, dose, and food matrix differ. A low-dose mulberry fruit extract reduced postprandial glucose and insulin responses in a randomized pilot study in unmedicated adults with type 2 diabetes [31], while a mulberry leaf combination containing fiber, vitamin D, and chromium altered postprandial glucometabolic responses but could not isolate every contribution of the mixture [32]. Clinical interpretation should stay product-specific.

Accordingly, isolated DNJ is treated as a defined compound, DNJ-enriched leaf extracts and twig alkaloid tablets as separately standardized preparations, fruit extracts as distinct botanical products, and teas or multi-ingredient formulations as food or combination interventions. A clinical result for one category is not assigned to another without chemical equivalence; a mixture cannot establish the contribution of mulberry alone [7,8,9,30,31,32,33,34,35,36,37].

### 4.3. Salacia Thiosugars

Species of Salacia contain sulfonium thiosugars such as salacinol, kotalanol, and related compounds that inhibit intestinal α-glucosidases. Earlier studies demonstrated antidiabetic activity of *Salacia reticulata* leaf extract in mice and assessed *Salacia chinensis* quality using α-glucosidase inhibitory activity as a bioactivity-guided criterion [38,39]. Their polarity and target location make local gastrointestinal pharmacology plausible. Synthetic modification of salacinol confirmed that side-chain length can increase potency against human intestinal maltase [14]. This structure-to-target connection distinguishes Salacia from extracts supported only by total phenolics or yeast enzyme screening.

Clinical meal-challenge studies support an acute effect. In people with type 2 diabetes, *Salacia oblonga* extract reduced postprandial glucose and insulin exposure after a carbohydrate-rich meal [20]. Randomized crossover studies in healthy volunteers also reported reductions in glycemic or insulinemic responses [40,41]. *Salacia chinensis* produced dose-dependent suppression of postprandial glucose in a randomized study and improved longer-term glycemic variables in a separate 12-week trial [42]. The mechanism is coherent, but extract composition, sponsorship, gastrointestinal effects, and replication across independent products should remain visible in interpretation.

Heterogeneity also occurs within Salacia: *S. oblonga*, *S. chinensis*, and *S. reticulata*, and their roots, stems, or leaves, cannot be treated as one intervention. Aqueous versus other extraction procedures, extraction ratios, salacinol/kotalanol/neokotalanol content, administered extract mass, and capsule, beverage, or biscuit matrices can change effective intestinal exposure. For example, acute *S. oblonga* doses of 240–480 mg [20] cannot be equated by extract mass alone with *S. chinensis* at 150–600 mg or 600 mg three times/day [42]. Unreported marker contents prevent a reliable cross-product potency ranking.

### 4.4. Berberine at the Incretin Interface

Berberine is poorly absorbed, making local intestinal exposure plausible, although effective human target-site concentrations remain unestablished; previous literature has described its progression from cellular pharmacology toward clinically relevant metabolic effects [43]. In human enteroendocrine NCI-H716 cells, berberine stimulated GLP-1 secretion through the bitter taste receptor TAS2R38 and a phospholipase C-dependent pathway; receptor blockade and knockdown reduced the response [5]. As a contextual preclinical example outside the selected English-language evidence corpus, a Chinese-language study in diabetic rats reported that short-term berberine increased intestinal GLP-1, reduced intestinal DPP-4, and preferentially lowered postprandial rather than fasting glucose, findings consistent with a possible local intestinal mechanism [44].

Metabolism may extend or redirect this mechanism. Berberrubine and palmatine increased GLP-1 production or glucose-stimulated secretion in GLUTag cells while alleviating oxidative and mitochondrial stress in a 2024 study [16]. These results support active-metabolite testing, but they do not by themselves demonstrate that the metabolites reach enteroendocrine cells at the effective concentrations in humans. The most informative clinical experiment would combine a standardized berberine formulation with intestinal or plasma exposure, active and total GLP-1, DPP-4 activity, gastric emptying, and postprandial glucose.

The TAS2R38–PLC and intestinal DPP-4 observations are cellular or animal findings, not established human mechanisms. Berberine-associated changes in glucose may also involve hepatic glucose production, insulin sensitivity, inflammation, and microbiota-related pathways [43,45]. The trials and models discussed here do not quantify the independent contribution of DPP-4 inhibition or GLP-1 secretion to any clinical glycemic effect.

### 4.5. Mangiferin, Anthocyanins, Guava Flavonols, and Food-Derived Peptides

Previous studies have reported DPP-4 inhibitory activity associated with *Mangifera indica* [46]. Mangiferin subsequently reduced circulating DPP-4 activity and improved glycemic, lipid, and β-cell-related endpoints in a high-fat diet/streptozotocin rat model [6]. A later comparison of berberine and mangiferin with pharmaceutical controls in diabetic metabolic-syndrome rats reported improvements across insulin resistance, inflammation, lipids, and β-cell preservation [45]. These multi-endpoint preclinical findings are suggestive, but causal DPP-4 dependence, free exposure, and selectivity require stronger confirmation.

Cyanidin 3,5-diglucoside was identified as a DPP-4 inhibitor in aronia juice [47], and flavonol glycosides from guava leaves inhibited DPP-4 in vitro [48]. These chemically anchored observations are more informative than extract docking, yet juice and leaf products contain many constituents and can act through carbohydrate digestion, transport, microbiota, or insulin signaling. A direct incretin claim should connect marker exposure to active GLP-1 preservation in vivo.

**Figure 2:**
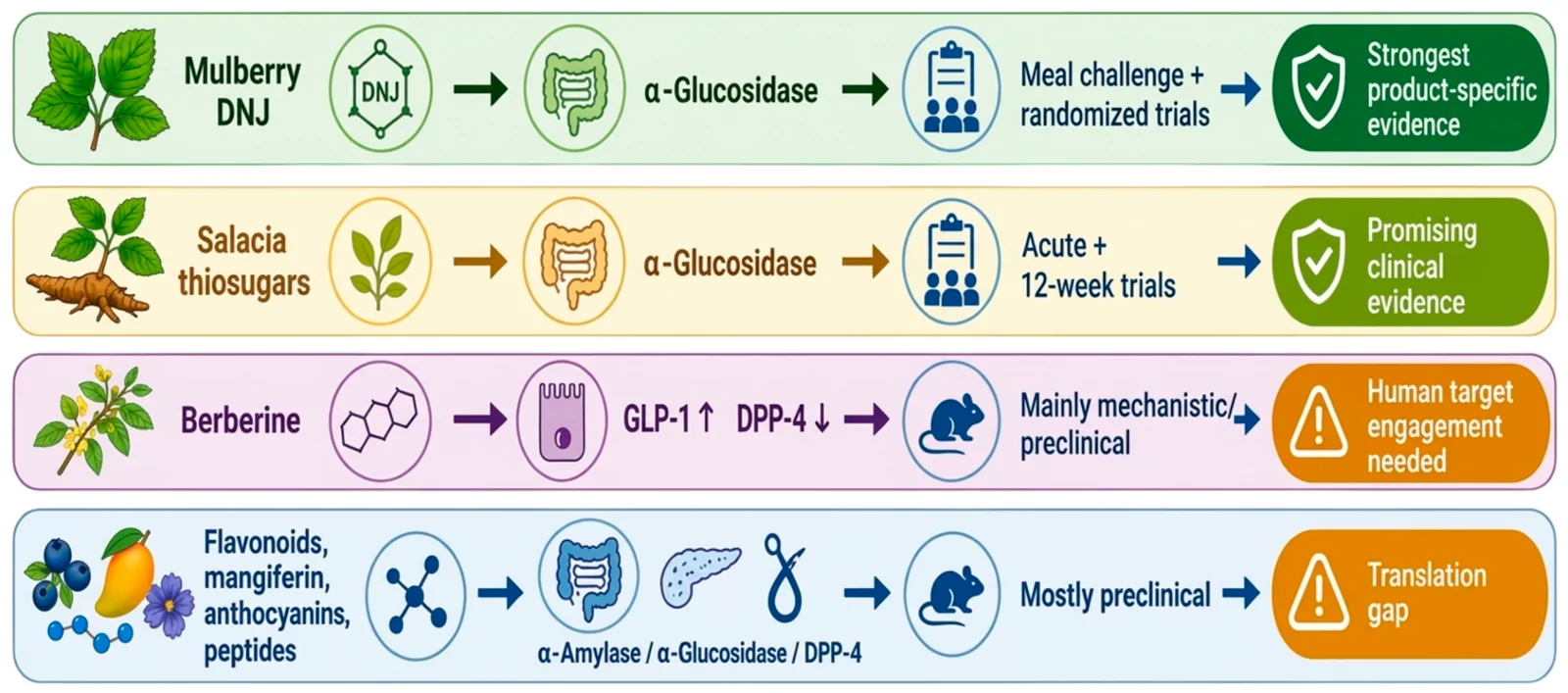
Chemistry-to-clinic evidence map for plant-derived postprandial glycemic modulators. Mulberry DNJ preparations and *Salacia* thiosugars currently have the strongest product-specific clinical support, whereas berberine and other phytochemical classes require additional human target-engagement evidence. This conceptual map distinguishes the type of evidence from the development requirements summarized in the tables; it is not a comparative efficacy estimate. Created in BioRender. Varut, M. (2026) https://BioRender.com/hf5tr6f.

Protein hydrolysates represent a different modality. A Palmaria palmata hydrolysate inhibited DPP-4 and influenced insulin, GLP-1, and GIP secretion in cellular systems, with acute animal glucose-tolerance testing used to extend the observation [49]. However, digestion can both create and destroy active peptides. Comparative work with food proteins showed that in vitro potency did not reliably predict plasma DPP-4 inhibition after oral administration [17]. Standardized gastrointestinal digestion, peptide identification, brush-border models, and in vivo exposure are therefore essential (Table 3 and Figure 2).

## 5. Human Evidence: Where Chemistry and Clinical Response Converge

Human evidence is strongest when the intervention is chemically characterized, administered with a defined carbohydrate challenge or for a prespecified treatment period, and evaluated against placebo or an active comparator. Acute meal studies answer whether a product changes glucose appearance under controlled conditions. Longer trials address durability and HbA1c but introduce adherence, background therapy, diet, and weight change. Continuous glucose monitoring can reveal time in range and meal-specific excursions that are missed by fasting glucose alone.

### 5.1. Mulberry Alkaloid Preparations

Mulberry twig alkaloid tablets have advanced beyond acute meal testing. A 24-week multicenter randomized double-blind double-dummy trial in 600 people with type 2 diabetes found HbA1c reduction non-inferior to acarbose, with fewer treatment-related and gastrointestinal adverse events in the mulberry group [8]. A separate randomized placebo-controlled multicenter study of 200 participants reported HbA1c changes of −0.80 percentage points (95% CI −0.98 to −0.62) with treatment and −0.09 (95% CI −0.27 to 0.09) with placebo after 16 weeks, together with improved fasting and postprandial endpoints [9]. An earlier smaller active-controlled trial provided preliminary support and highlighted the need for the larger studies [33].

The program has continued to clinically relevant phenotyping. In people inadequately controlled on oral therapy, a 2026 randomized study using flash glucose monitoring compared mulberry twig alkaloids with canagliflozin; both improved several glycemic metrics, with differences in selected postprandial excursions. This small open-label study does not establish overall equivalence or superiority [34]. A small three-arm study in insulin-treated patients used continuous glucose monitoring to compare add-on mulberry alkaloids, metformin, and insulin alone [35]. These findings add product-specific observations, although open questions include long-term cardiovascular and renal outcomes, external replication, generalizability, and detailed constituent-exposure relationships.

Not every mulberry product has the same evidence. A randomized crossover study of a low-dose fruit extract in 24 unmedicated adults with type 2 diabetes showed acute postprandial effects [31], while mulberry leaf plus corn silk reduced selected glucose endpoints mainly in a subgroup in a 2025 crossover trial [36]. A mulberry leaf and water chestnut tea also reduced postprandial glucose in borderline diabetes [37]. These studies may support food-format development, but they cannot be pooled directly with standardized twig alkaloid tablets.

### 5.2. Salacia Extracts

Salacia provides a second coherent mechanism-to-human example. Acute randomized crossover studies in healthy participants and people with type 2 diabetes generally showed reduced postprandial glucose and/or insulin exposure after carbohydrate-rich meals [20,40,41]. Dose–response and 12-week data with *S. chinensis* strengthened the association between an α-glucosidase-inhibitory extract and glycemic endpoints [42]. A randomized trial in overweight or obese adults found attenuation of glycemic indices and changes in selected gastrointestinal peptides, but no overall GLP-1 area-under-the-curve difference, illustrating that α-glucosidase inhibition does not guarantee a uniform incretin response [50].

A triple-blind crossover trial of *Salacia reticulata* extract biscuits in people with type 2 diabetes tested a food-format intervention over longer periods [51]. Such formats are attractive for meal-linked dosing but complicate blinding, stability, and control of background carbohydrate intake. Future studies should report salacinol, kotalanol, and related marker contents, enzyme-inhibition potency per dose, adherence, gastrointestinal events, and whether the clinical product matches the material used in mechanistic assays.

### 5.3. Mixed Botanical Products and Negative Translation

Tangzhiqing, a multi-herb Chinese product, was evaluated in an eight-period crossover study using glucose, sucrose, maltose, maltodextrin, and starch challenges. It reduced responses to selected hydrolyzable carbohydrates but not free glucose, supporting a digestive-enzyme mechanism and demonstrating the value of substrate-discrimination designs [52]. The non-linear dose response also cautions against assuming that more enzyme inhibition produces proportionally greater benefit.

Negative or discordant studies are equally informative. A 2026 randomized crossover study of a polyphenol-rich sugarcane extract found in vitro inhibition of human sucrase and weaker effects on maltase/isomaltase, yet did not reduce postprandial glucose or insulin responses in healthy adults [53]. The null result limits efficacy claims for that product, dose, population, and starch-containing meal; it does not prove absence of all biological activity or equivalence to placebo. Differences in dose, food matrix, effective luminal concentration, inhibitor timing, and compensatory physiology should be investigated rather than treating the clinical null result as an anomaly.

The trial used liquid extract at 0.5% or 5% with a 50 g-carbohydrate meal and 180 min sampling. Glucose iAUC did not differ significantly from the corresponding controls (*p* = 0.49 and 0.22); human sucrase IC50 was 425.8 ± 18.7 μg/mL, while the extract activated rather than inhibited human α-amylases in vitro [53]. The small sample and substrate mismatch limit generalization. Null HbA1c findings in the 12-week DNJ-enriched leaf study [7] and absent overall GLP-1 AUC differences with *S. chinensis* [50] likewise separate acute glycemic effects from sustained efficacy and incretin mechanisms (Table 4).

## 6. Why In Vitro Potency Often Fails to Translate

An IC50 is conditional on enzyme concentration, substrate identity and concentration, incubation time, buffer, pH, detector, and inhibitor solubility. It is not an intrinsic clinical dose. For competitive inhibitors, the apparent potency changes with substrate concentration. For mixed or non-competitive inhibitors, different kinetic components matter. Colored extracts can absorb at assay wavelengths; tannins can precipitate proteins; and reducing compounds can alter coupled detection systems. Orthogonal readouts and extract blanks are therefore required.

Enzyme-source mismatch is especially consequential for α-glucosidase. Yeast screening is acceptable for discovery but should be followed by mammalian and preferably human maltase-glucoamylase and sucrase-isomaltase assays. The Vauquelinia example, in which potency differed substantially between yeast and mammalian enzymes, shows that the rank order can change [12]. For α-amylase, both salivary and pancreatic isoforms should be considered, and the inhibitor should be tested against realistic starch substrates with physiologically plausible contact times.

**Figure 3:**
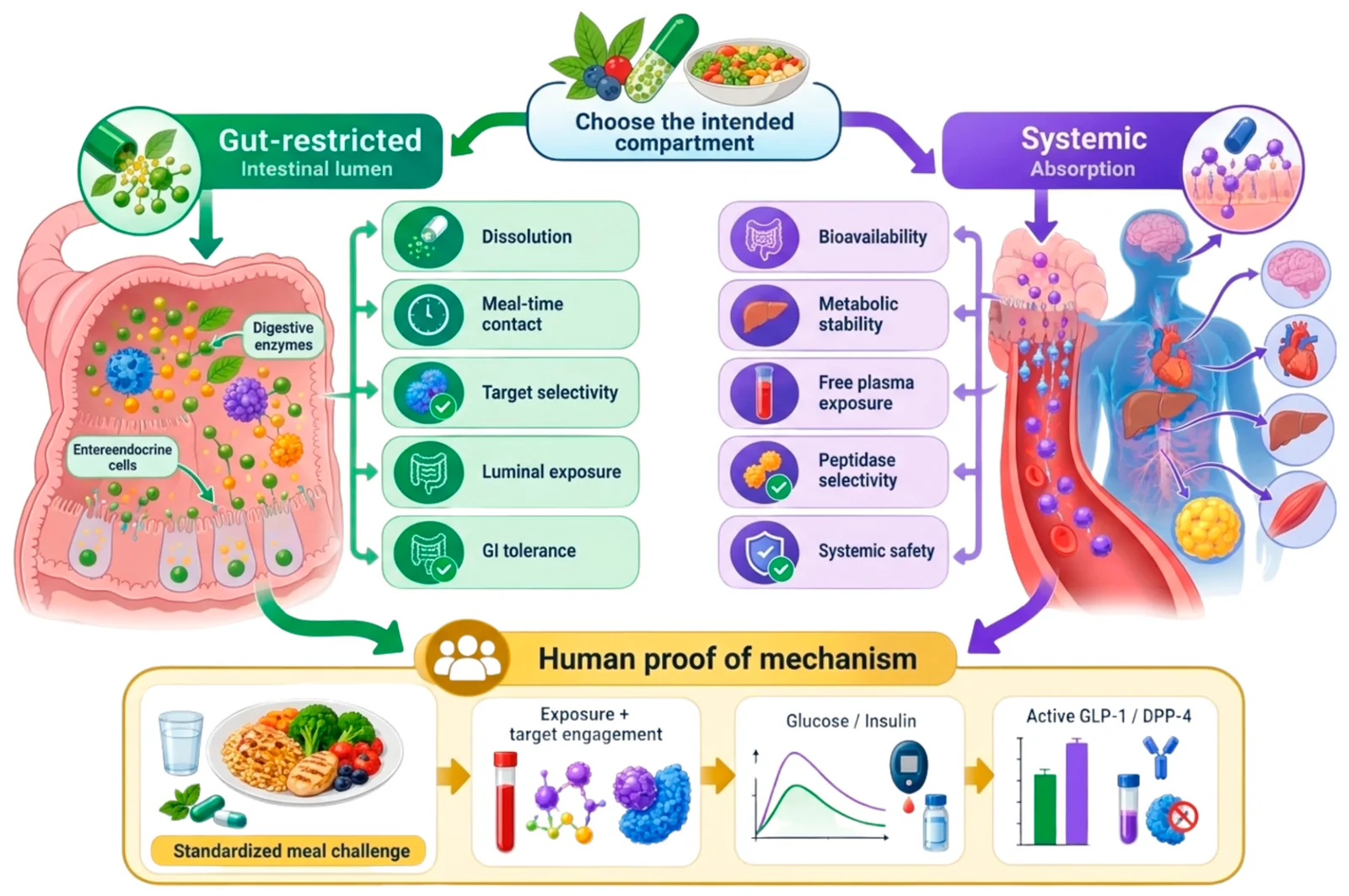
Compartment-led development of plant-derived postprandial glycemic modulators. Gut-restricted candidates are optimized for luminal dissolution, meal-time contact, target selectivity, and gastrointestinal tolerability, whereas systemic candidates require bioavailability, metabolic stability, free plasma exposure, peptidase selectivity, and systemic safety. Both pathways require exposure-linked human proof of mechanism. The two paths depict distinct experimental decisions rather than observed clinical equivalence. Created in BioRender. Varut, M. (2026) https://BioRender.com/hskesy9.

The luminal concentration is determined by dose, dissolution, meal volume, binding to food, and timing. A poorly absorbed compound can be advantageous for an intestinal enzyme target, but only if it dissolves and remains active. For DPP-4, the relevant site must be specified. Local intestinal inhibition is a preclinical hypothesis even when plasma activity is unchanged, as proposed in rats for berberine [44]. Systemic DPP-4 claims require free plasma or tissue exposure, enzyme activity, and active GLP-1 preservation (Figure 3).

Finally, the body compensates. Slower carbohydrate hydrolysis can alter gastric emptying, insulin, glucagon, GLP-1, GIP, amylin, substrate oxidation, and later hunger. An acute reduction in the early glucose peak may be offset by delayed appearance later in the sampling window. Meal studies should therefore sample long enough to capture the full excursion and should report glucose and insulin iAUC, peak timing, late glucose, active and total incretins, and gastrointestinal symptoms.

## 7. Safety, Interactions, and Product Quality

Gastrointestinal effects are mechanism-linked for carbohydrate-hydrolysis inhibitors. Undigested carbohydrate increases osmotic load and fermentation, so flatulence, abdominal discomfort, and diarrhea should be recorded with standardized definitions and related to meal composition. In the 24-week mulberry twig trial, gastrointestinal events occurred in 12.2% versus 24.6% with acarbose, and treatment-related events in 15.3% versus 28.4%; no hypoglycemia was reported in either group [8], but it should be confirmed across doses, diets, and longer exposure. Reduced symptoms must not result from inadequate enzyme inhibition.

In the acute *S. oblonga* crossover trial, gastrointestinal symptoms were reported after control, 240 mg, and 480 mg by 2, 8, and 12 participants, respectively, and lasted no more than 24 h [20]. These crossover counts are not independent parallel-group event rates. The 12–24-week studies discussed here cannot establish rare-event or multi-year safety; this limitation also applies to food formulations and products with different chemical specifications.

The mechanism of a purely local digestive inhibitor suggests a low intrinsic hypoglycemia risk when used alone, but risk can increase when postprandial modulators are combined with insulin or insulin secretagogues. Combination trials should prespecify glucose monitoring and hypoglycemia reporting; the absence of events in monotherapy cannot be generalized to insulin-treated populations. Products that stimulate GLP-1 or inhibit DPP-4 may have broader gastrointestinal or immune effects, and non-selective inhibition of related peptidases is undesirable. Berberine has clinically demonstrated enzyme-interaction potential: in a crossover study of 17 healthy completers, 300 mg three times daily for 14 days reduced CYP2D6, CYP2C9, and CYP3A4 activity; midazolam exposure increased by about 40% [54]. This supports medication review for susceptible substrates, especially drugs with narrow therapeutic margins. It does not establish the magnitude of every proposed herb–drug interaction.

Quality specifications should include botanical identity, plant part, extraction solvent, drug-to-extract ratio, relevant active markers, enzyme potency per unit dose, contaminants, microbial limits, residual solvents, and stability. For mulberry, DNJ or a defined alkaloid panel is mechanistically relevant. For Salacia, salacinol, kotalanol, and related thiosugars should be quantified. Total phenolics alone are not an adequate release specification for a product developed as an α-glucosidase or DPP-4 inhibitor.

## 8. A Development Framework for Molecules and Standardized Products

Development should start by choosing the intended compartment. A gut-restricted product can be optimized for dissolution, meal-time contact, and selectivity among digestive enzymes without pursuing high plasma exposure. A systemic DPP-4 inhibitor requires absorption, metabolic stability, peptidase selectivity, and conventional systemic toxicology. A GLP-1 secretagogue should reach the relevant enteroendocrine sensors and demonstrate receptor-dependent secretion rather than only elevated circulating GLP-1 after delayed digestion.

**Figure 4:**
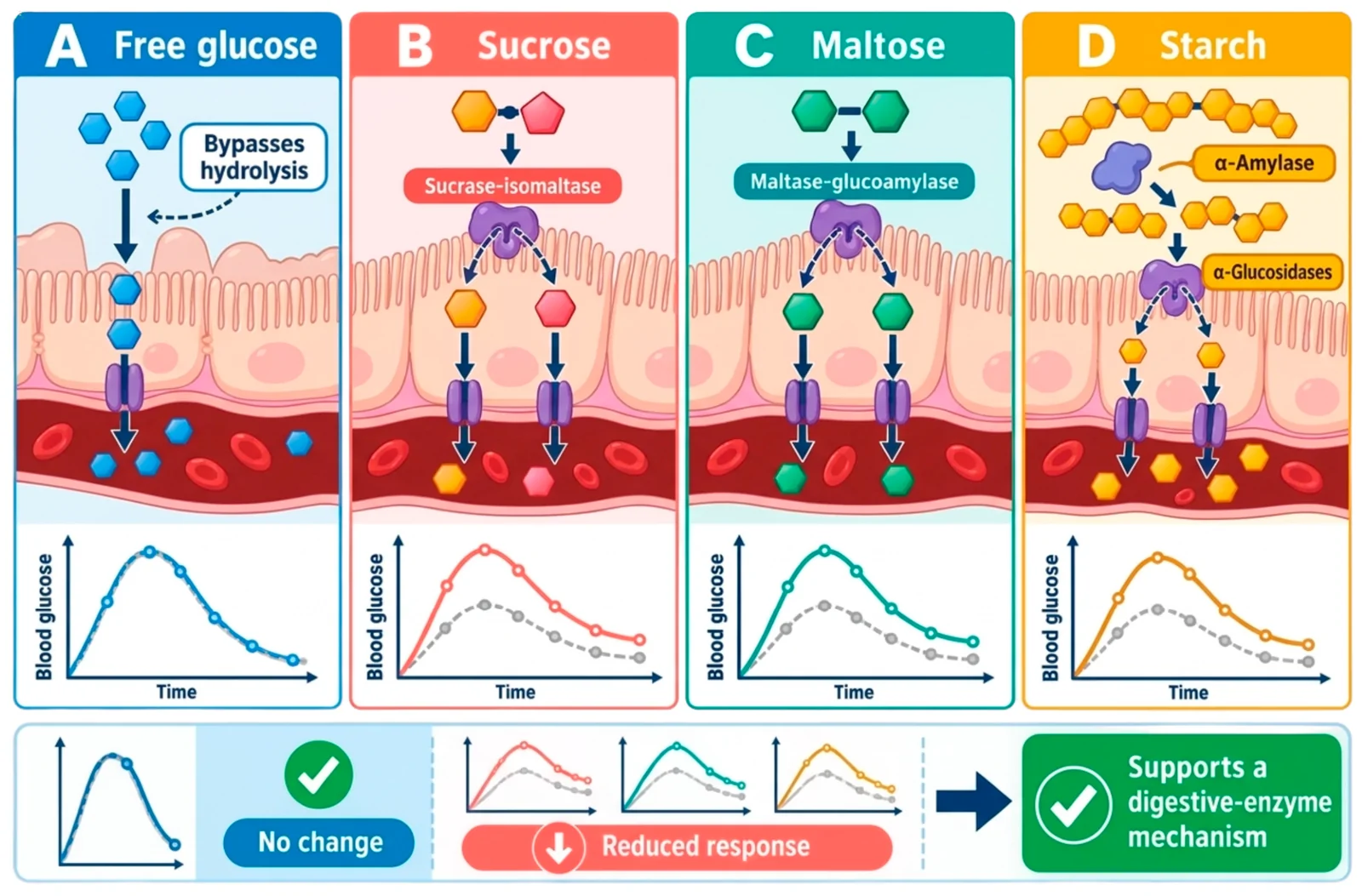
Target-matched carbohydrate challenges for mechanism discrimination. Free glucose bypasses hydrolysis, whereas sucrose, maltose, and starch probe sucrase–isomaltase, overlapping intestinal maltase activities, and sequential α-amylase/α-glucosidase activity. Selective attenuation of responses to hydrolyzable carbohydrates supports a digestive-enzyme mechanism. These are functional substrate probes, not enzyme-complex-specific tests. Created in BioRender. Varut, M. (2026) https://BioRender.com/64cnko4.

The carbohydrate challenge should match the target. Sucrose tests sucrase activity; maltose tests maltase; starch requires amylase plus downstream α-glucosidases; and free glucose bypasses hydrolysis. The Tangzhiqing study illustrates how a multi-substrate design can distinguish digestive inhibition from downstream insulin sensitization [52]. This approach should be used earlier, in both animal and human proof-of-mechanism studies (Figure 4).

A minimum clinical development sequence would include: an acute randomized crossover meal challenge with exposure and gastrointestinal measurements; a dose-ranging study to define the efficacy-tolerability window; and a longer randomized trial using the same standardized product. Continuous glucose monitoring can identify which meals and time windows respond, while HbA1c establishes durability. Mechanistic biomarkers should be prespecified and limited to those that can discriminate among α-amylase, α-glucosidase, GLP-1 secretion, and DPP-4 inhibition.

The checklist in Table 5 is an author-developed synthesis for this narrative review, informed by the assay-to-clinic discrepancies and product-specific trial designs discussed above [7,8,10,11,12,17,20,52,53]. It is neither a validated scoring instrument nor an externally endorsed drug-development standard; the domains should be tested prospectively (Table 5).

## 9. Conclusions

Plant-derived control of postprandial glycemia is most credible when mechanism and compartment align. Flavonoids provide valuable structure–activity information for α-amylase, α-glucosidase, and DPP-4, but assay-dependent potency and extensive metabolism limit direct therapeutic inference. Preclinical berberine studies suggest a possible intestinal link between bitter-taste-receptor-mediated GLP-1 secretion and local DPP-4 effects, whereas mangiferin, anthocyanins, guava flavonols, and protein hydrolysates remain primarily preclinical.

Mulberry iminosugar preparations and Salacia thiosugars currently provide the most developed product-specific human evidence among the candidates considered. Standardized mulberry twig alkaloid tablets have shown placebo-controlled efficacy and non-inferiority to acarbose, while Salacia extracts repeatedly reduced acute postprandial responses and have some longer-term support. These findings cannot be generalized to all mulberry or Salacia products, much less to all antidiabetic plants. Product identity is part of the mechanism.

Future progress depends on replacing potency lists with target-relevant enzyme panels, substrate-discrimination studies, luminal or systemic exposure measurements, active incretin assays, and controlled trials using a locked chemical specification. Negative translation should be reported and used to refine the model. Only a limited number of rigorously characterized preparations have replicated controlled human findings, and replication across independent batches and populations remains incomplete. The same standard applies to all candidates: in vitro potency alone is insufficient, acute postprandial effects do not establish durable therapeutic benefit, and clinical glucose lowering does not by itself prove the proposed molecular target.

## Figures and Tables

**Table 1:** Operational evidence categories for this narrative synthesis.

Category	Operational Definition	Example and Allowable Inference
H Hypothesis generating	Yeast-only enzyme screening, docking, or indirect mechanism without target-relevant confirmation	A screening hit prioritizes follow-up; it cannot establish human intestinal enzyme inhibition.
B Biochemical or cellular	Chemically identified intervention tested in relevant mammalian/human enzyme or cell systems, with concentration–response or pathway controls	Human α-amylase assays [4] or NCI-H716 receptor knockdown [5]; activity is demonstrated only in that system.
A Animal mechanistic	In vivo animal exposure with a target-related biomarker and a metabolic endpoint	Mangiferin rat measurements [6]; mechanistic association remains preclinical.
C1 Human target compatible	Controlled human challenge with a characterized product and target-matched substrate or biomarker	DNJ rice challenge [7]; compatible with digestive inhibition, but not direct molecular target engagement.
C2 Human clinical outcome	Controlled trial with glucose or HbA1c outcomes, without direct demonstration of the proposed molecular target	Twig alkaloid HbA1c trials [8,9]; product-specific clinical effect, with mechanistic uncertainty.

**Table 2:** Target-specific evidence requirements for plant-derived control of postprandial glycemia. These are proposed evidence requirements, not findings established for every candidate.

Target or Process	Most Relevant Assays	Exposure Requirement	Frequent Interpretive Error
α-Amylase	Human salivary and pancreatic enzymes; kinetic mode; realistic starch substrate; meal matrix [4,10,11]	High luminal concentration during starch digestion may be sufficient	Extrapolating from porcine enzyme or chromogenic substrate alone
Intestinal α-glucosidases	Human or mammalian maltase-glucoamylase and sucrase-isomaltase; substrate-specific kinetics [12,13,14]	Brush-border exposure during the relevant disaccharide/oligosaccharide load	Using yeast α-glucosidase as a direct clinical surrogate
GLP-1 secretion	Enteroendocrine cells or organoids; receptor knockdown; active and total GLP-1; meal challenge [5,15,16]	Local intestinal exposure to secretagogue or metabolite	Attributing delayed nutrient delivery to direct L-cell stimulation
DPP-4 inhibition	Human recombinant and cell-surface enzyme; active GLP-1 [17,18,19]	Local intestinal or systemic free concentration consistent with potency	Treating docking or weak micromolar inhibition as systemic target engagement
Clinical postprandial control	Randomized standardized-meal challenge; glucose and insulin iAUC; GLP-1/GIP; CGM [7,8,20]	Same chemically standardized product used in mechanistic studies	Generalizing an acute meal effect to durable HbA1c efficacy

iAUC, incremental area under the curve; CGM, continuous glucose monitoring. Exposure requirements are the authors’ interpretation of the cited experimental designs; prospective confirmation is required. Selectivity against related peptidases is an additional proposed safety requirement.

**Table 3:** Mechanistic examples at the carbohydrate-digestion and incretin-DPP-4 interface. SAR, structure–activity relationship; PLC, phospholipase C; TAS2R38, taste receptor type 2 member 38. Evidence categories are defined in Table 1.

Candidate	Model and Tested Exposure	Principal Finding	Evidence Level and Limit
Selected flavonoids	Human/mammalian enzyme assays [4,13]; paired yeast/rat intestinal assays [12]	Benzoylated quercetin glycoside: IC50 30 μM (yeast) versus 437 μM (rat intestine) [12]	B; yeast-only findings are H. No cross-assay clinical ranking.
Mulberry DNJ/iminosugar fractions	DNJ-enriched leaf extract: 3–9 mg DNJ in a human rice challenge [7]; twig tablets are a separate preparation [8]	Dose-related acute glucose attenuation [7]; quantitative trial outcomes in Table 4	C1 for target-compatible meal effects; C2 for HbA1c. Direct human enzyme occupancy not established.
Salacinol/kotalanol; Salacia species	Human intestinal maltase assays [14]; extract doses 150–600 mg in humans [42]	Side-chain modification increased maltase inhibitory potency [14]; exact absolute potency values for individual analogues were not consistently reported in the accessible primary source.	B for scaffolds; C1/C2 for specific extracts. Analogue results do not establish botanical efficacy.
Berberine and metabolites	Human NCI-H716 cells [5]; diabetic rats, 30–120 mg/kg/day [44]	Receptor knockdown attenuated cellular secretion [5]; intestinal DPP-4/GLP-1 changes in rats [44]	B/A; human target engagement absent. Rat study is contextual non-English evidence.
Mangiferin	High-fat diet/streptozotocin rats; oral mangiferin 40 mg/kg during weeks 4–10 [6]	Reduced circulating DPP-4 activity and altered metabolic endpoints [6,45]	A; association does not establish causal DPP-4 dependence.
Myricetin and flavonoid glycosides	Purified DPP-4 screen at 200 μM; concentration–response assays [18]	IC50: myricetin 156.29 ± 1.18 μM; cyanidin 3-O-glucoside 81.05 ± 4.14 μM [18]	B; enzyme origin, assay conditions, and achievable free exposure constrain translation.
Aronia anthocyanin; guava flavonols	Biochemical DPP-4 assays [47,48]; guava ethanolic leaf extract concentration–response	Guava extract IC50 380 μg/mL [48];	B; extract mass is not a purified-compound concentration or a clinical dose.
Palmaria and food-protein hydrolysates	Biochemical/cellular secretion assays and acute animal testing [17,49]; relevant human exposure data were not reported	In vitro DPP-4 inhibition did not consistently predict in vivo inhibition [17]	B/A; digestive stability, peptide identity, and human exposure unresolved.

**Table 4:** Selected controlled human studies with chemically or mechanistically informative plant products.

Intervention and Sample	Dose, Comparator and Duration	Quantitative Outcome	Safety and Interpretation
Twig alkaloid tablets; T2DM; *n* = 600 randomized (360/240) [8]	SZ-A 50 mg three times/day for 4 weeks, then 100 mg three times/day; acarbose 50 mg three times/day; 24 weeks	HbA1c: −0.93 vs. −0.87 pp; adjusted difference −0.05 pp (95% CI −0.18 to 0.07); non-inferiority margin 0.30 pp	GI events 12.2% vs. 24.6%; treatment-related events 15.3% vs. 28.4%. Product-specific 24-week evidence.
Twig alkaloid tablets; T2DM; *n* = 200 (100/100) [9]	SZ-A vs. placebo; double-blind; 16 weeks.	HbA1c −0.80 pp (95% CI −0.98 to −0.62) vs. −0.09 (−0.27 to 0.09)	Supports a treatment effect in this trial; AE/GI rates differed. Does not establish broad efficacy.
Twig alkaloids; T2DM; *n* = 60 randomized, 55 completed [34]	50 mg three times/day vs. canagliflozin 100 mg/day; open-label; 12 weeks	HbA1c decreased 0.93 vs. 0.94 pp; no significant between-group difference. Selected meal excursions favored SZ-A.	One SZ-A stomachache withdrawal; one comparator hypoglycemia withdrawal. No proof of equivalence from non-significance.
DNJ-enriched leaf extract; impaired glucose metabolism; *n* = 12 [7]	0, 3, 6 or 9 mg DNJ; placebo-controlled four-period crossover; rice challenge	Acute dose-related attenuation of postprandial glucose was reported [7]; between-group iAUC estimates with confidence intervals were not available for extraction. A separate 12-week study *(n* = 76; 6 mg three times daily) found no between-group difference in HbA1c.	Acute and durable outcomes differ. No serious adverse events reported; product not interchangeable with twig tablets.
*S. chinensis* extract [42]	150, 300 or 600 mg vs. placebo, acute crossover; separate 600 mg three times/day, 12-week trial (corrected report)	Dose-related acute suppression and longer-term improvement in glycemic variables were reported; however, numerical between-group effect estimates and corresponding confidence intervals were not available for extraction from the primary report [42].	Evidence remains extract-specific; no pooled estimate.
*S. oblonga*; T2DM; *n* = 66 analyzed [20]	240 or 480 mg vs. control liquid meal; double-blind crossover; 180 min sampling	Glucose positive AUC lower by 14%/22%; insulin positive AUC lower by 14%/19%, respectively	GI symptoms: 2 after control, 8 after 240 mg, 12 after 480 mg; ≤24 h. Acute outcome only.
Tangzhiqing combination; *n* = 36 healthy volunteers (12 per dose group) [52]	3, 6 or 9 tablets (0.64 g each); eight-period carbohydrate challenges; 120 min sampling	With 6 tablets, starch glucose iAUC: 109.15 ± 55.92 vs. 57.68 ± 46.09 mmol·min/L (mean ± SD; *p* = 0.01). No reduction with free glucose.	Two mild events, considered unrelated; no serious events. No monotonic dose response; acute substrate effects do not prove a molecular target.
Polyphenol-rich sugarcane extract; healthy adults [53]	0.5% or 5% liquid extract; matched controls; 50 g-carbohydrate meal; 180 min. Abstract *n* = 12; figure *n* = 13.	No significant glucose iAUC reduction (*p* = 0.49/0.22) or insulin iAUC reduction (*p* = 0.42/0.07)	Sample-size discrepancies limit interpretation; adverse-event rates and corresponding confidence intervals were not available for extraction. A null result should not be interpreted as evidence of equivalence.

T2DM, type 2 diabetes mellitus; GI, gastrointestinal; DNJ, 1-deoxynojirimycin; pp, percentage points.

**Table 5:** Translational checklist for a plant-derived postprandial glucose modulator.

Domain	Required Decision Information	Preferred Evidence	Reason to Pause Development
Product chemistry	Active marker(s), batch fingerprint, potency per dose, stability	Validated quantitative method linked to bioactivity	Unknown active fraction or major batch drift
Target assay	Correct human/mammalian enzyme, substrate, kinetics, selectivity, interference controls	Orthogonal biochemical and cell-surface confirmation	Yeast enzyme or docking as the only target evidence
Compartment exposure	Luminal dissolution and concentration, or systemic free concentration	Meal-simulated dissolution, intestinal model, PK and metabolite data	Effective assay concentration cannot be reached
Mechanistic human test	Target-matched carbohydrate challenge; glucose/insulin iAUC; active GLP-1/DPP-4 if claimed	Randomized crossover with adequate sampling and identical product	No substrate specificity or no target-relevant biomarker
Durability and safety	HbA1c/CGM, GI tolerability, hypoglycemia, interactions, adherence	Randomized longer trial with prespecified safety	Benefit depends on intolerable carbohydrate malabsorption
Reproducibility	Independent population and manufacturing batch	Replication with locked specification	Clinical product differs chemically from mechanistic material

The pathway should be declared as gut-restricted or systemic before dose and formulation optimization.

## Data Availability

No new data were created or analyzed in this study. Data sharing is not applicable to this article.

## Supplementary Materials

The following supporting information can be downloaded at: https://www.mdpi.com/article/10.3390/ph19101546/s1.

## Abbreviations

The following abbreviations are used in this manuscript:

CGM	Continuous glucose monitoring
DNJ	1-Deoxynojirimycin
DPP-4	Dipeptidyl peptidase-4
GIP	Glucose-dependent insulinotropic polypeptide
GLP-1	Glucagon-like peptide-1
GI	Gastrointestinal
iAUC	Incremental area under the curve
PLC	Phospholipase C
T2DM	Type 2 diabetes mellitus
TAS2R38	Taste receptor type 2 member 38
