# Supplementary material for: Plant-Derived Control of Postprandial Glycemia: α-Amylase, α-Glucosidase, and the Incretin-DPP-4 Interface
Source: Pharmaceuticals (Basel). 2026 Sep 30;19(10):1546. doi: 10.3390/ph19101546 (PMC13708452; doi:10.3390/ph19101546)
Supplement: Supplementary file 1 [file pharmaceuticals-19-01546-s001.zip › pharmaceuticals-4586422-supplementary.pdf]

Supplementary Table S1. Full electronic search strategies used for the literature search

Database	Full search strategy	Search date	Records retrieved
**PubMed/MEDLINE**	(("alpha-amylase"[Title/Abstract] OR "α-amylase"[Title/Abstract] OR "alpha-glucosidase"[Title/Abstract] OR "α-glucosidase"[Title/Abstract] OR "maltase-glucoamylase"[Title/Abstract] OR "sucrase-isomaltase"[Title/Abstract] OR "DPP-4"[Title/Abstract] OR "DPP-IV"[Title/Abstract] OR "GLP-1"[Title/Abstract] OR "glucagon-like peptide-1"[Title/Abstract] OR incretin[Title/Abstract]) AND (plant[Title/Abstract] OR phytochemical[Title/Abstract] OR botanical[Title/Abstract] OR "natural product"[Title/Abstract] OR flavonoid[Title/Abstract] OR polyphenol[Title/Abstract] OR alkaloid[Title/Abstract] OR iminosugar[Title/Abstract] OR "1-deoxynojirimycin"[Title/Abstract] OR mulberry[Title/Abstract] OR Morus[Title/Abstract] OR Salacia[Title/Abstract] OR salacinol[Title/Abstract] OR kotalanol[Title/Abstract] OR berberine[Title/Abstract] OR berberrubine[Title/Abstract] OR palmatine[Title/Abstract] OR mangiferin[Title/Abstract] OR anthocyanin[Title/Abstract] OR guava[Title/Abstract] OR Psidium[Title/Abstract] OR "food-derived peptide"[Title/Abstract] OR "protein hydrolysate"[Title/Abstract])) OR (("postprandial glucose"[Title/Abstract] OR "postprandial glycemia"[Title/Abstract] OR "postprandial glycaemia"[Title/Abstract] OR "meal challenge"[Title/Abstract] OR "continuous glucose monitoring"[Title/Abstract] OR "flash glucose monitoring"[Title/Abstract]) AND (plant[Title/Abstract] OR phytochemical[Title/Abstract] OR botanical[Title/Abstract] OR "natural product"[Title/Abstract] OR flavonoid[Title/Abstract] OR polyphenol[Title/Abstract] OR alkaloid[Title/Abstract] OR iminosugar[Title/Abstract] OR "1-deoxynojirimycin"[Title/Abstract] OR mulberry[Title/Abstract] OR Morus[Title/Abstract] OR Salacia[Title/Abstract] OR salacinol[Title/Abstract] OR kotalanol[Title/Abstract] OR berberine[Title/Abstract] OR berberrubine[Title/Abstract] OR palmatine[Title/Abstract] OR mangiferin[Title/Abstract] OR anthocyanin[Title/Abstract] OR guava[Title/Abstract] OR Psidium[Title/Abstract] OR "food-derived peptide"[Title/Abstract] OR "protein hydrolysate"[Title/Abstract]))	19 August 2026	**82**

**Scopus**	TITLE-ABS-KEY(("alpha-amylase" OR "α-amylase" OR "alpha-glucosidase" OR "α-glucosidase" OR "maltase-glucoamylase" OR "sucrase-isomaltase" OR "DPP-4" OR "DPP-IV" OR "GLP-1" OR "glucagon-like peptide-1" OR incretin) AND (plant OR phytochemical OR botanical OR "natural product" OR flavonoid OR polyphenol OR alkaloid OR iminosugar OR "1-deoxynojirimycin" OR mulberry OR Morus OR Salacia OR salacinol OR kotalanol OR berberine OR berberrubine OR palmatine OR mangiferin OR anthocyanin OR guava OR Psidium OR "food-derived peptide" OR "protein hydrolysate")) OR TITLE-ABS-KEY(("postprandial glucose" OR "postprandial glycemia" OR "postprandial glycaemia" OR "meal challenge" OR "continuous glucose monitoring" OR "flash glucose monitoring") AND (plant OR phytochemical OR botanical OR "natural product" OR flavonoid OR polyphenol OR alkaloid OR iminosugar OR "1-deoxynojirimycin" OR mulberry OR Morus OR Salacia OR salacinol OR kotalanol OR berberine OR berberrubine OR palmatine OR mangiferin OR anthocyanin OR guava OR Psidium OR "food-derived peptide" OR "protein hydrolysate"))	19 August 2026	**76**
**Web of Science Core Collection**	TS=(("alpha-amylase" OR "α-amylase" OR "alpha-glucosidase" OR "α-glucosidase" OR "maltase-glucoamylase" OR "sucrase-isomaltase" OR "DPP-4" OR "DPP-IV" OR "GLP-1" OR "glucagon-like peptide-1" OR incretin) AND (plant OR phytochemical OR botanical OR "natural product" OR flavonoid OR polyphenol OR alkaloid OR iminosugar OR "1-deoxynojirimycin" OR mulberry OR Morus OR Salacia OR salacinol OR kotalanol OR berberine OR berberrubine OR palmatine OR mangiferin OR anthocyanin OR guava OR Psidium OR "food-derived peptide" OR "protein hydrolysate")) OR TS=(("postprandial glucose" OR "postprandial glycemia" OR "postprandial glycaemia" OR "meal challenge" OR "continuous glucose monitoring" OR "flash glucose monitoring") AND (plant OR phytochemical OR botanical OR "natural product" OR flavonoid OR polyphenol OR alkaloid OR iminosugar OR "1-deoxynojirimycin" OR mulberry OR Morus OR Salacia OR salacinol OR kotalanol OR berberine OR berberrubine OR palmatine OR mangiferin OR anthocyanin OR guava OR Psidium OR "food-derived peptide" OR "protein hydrolysate"))	19 August 2026	**62**

Note: No language filter was applied during the database searches. Eligibility was subsequently restricted to English-language publications during the screening process. Searches covered each database from inception to 19 August 2026. The three database searches yielded 220 records before deduplication; 35 duplicates were removed, leaving 185 unique records for title and abstract screening.
